# The Efficacy of Telepsychiatry in Addiction Patients: A Systematic Review

**DOI:** 10.7759/cureus.38133

**Published:** 2023-04-25

**Authors:** Hari Krishna Kamma, Mohammad Alabbas, Mohammad Elashahab, Naushad Abid, Sara Manaye, Kaaviya Cheran, Chinmayee Murthy, Elisa A Bornemann, Ana P Arcia Franchini

**Affiliations:** 1 Psychiatry, California Institute of Behavioral Neurosciences & Psychology, Fairfield, USA; 2 Cardiology/Internal Medicine, University of Debrecen, Debrecen, HUN; 3 Radiology, California Institute of Behavioral Neurosciences & Psychology, Fairfield, USA; 4 Rheumatology, King Faisal University, Hofuf, SAU; 5 Medicine, California Institute of Behavioral Neurosciences & Psychology, Fairfield, USA; 6 General Medicine, California Institute of Behavioral Neurosciences & Psychology, Fairfield, USA; 7 Internal Medicine, California Institute of Behavioral Neurosciences & Psychology, Fairfield, USA; 8 Medicine/Surgery, Universidad Latina de Panama, Panama City, PAN; 9 Internal Medicine/Neurology, California Institute of Behavioral Neurosciences & Psychology, Fairfield, USA; 10 Research, California Institute of Behavioral Neurosciences & Psychology, Fairfield, USA

**Keywords:** addiction therapy, substance addiction, addiction psychiatry, tele-health, substance use disorder (sud), tele psychiatry

## Abstract

Psychiatry is one of the many medical subspecialties that have benefited from the advent of telemedicine. Substance abuse treatment via telepsychiatry expeditiously increased with the start of the pandemic and has brought changes to its rules and regulations. In this study, we focused on the prognosis of substance abuse patients treated with telepsychiatry, the various changes that occurred during the pandemic, and the difficulties faced by clinicians using telepsychiatry. PubMed and Google Scholar were searched for relevant articles between January 2010 and July 2022 using both broad and narrow keywords in addition to the MeSH (Medical Subject Heading) approach. The total number of records found was 765. Strict criteria for inclusion and exclusion ensured that only relevant information was collected. After removing duplicates, irrelevant studies, and research that did not meet the inclusion criteria, we were left with 373 studies from both electronic databases. From those, we ultimately retrieved 35 studies, which were subjected to a thorough content search and quality evaluation with the help of specialized instruments, and a total of 19 papers were included in our systematic review. We concluded that telepsychiatry use for substance abuse patients increased during the pandemic, and the prognosis of these patients treated with telepsychiatry was similar to that of in-person treatment. However, a combination of telepsychiatry with in-person sessions showed much better results.

## Introduction and background

Psychiatry is one of the many medical subspecialties that have benefited from the advent of telemedicine. Telepsychiatry refers to the use of electronic communication technologies to provide psychiatric services such as assessment, diagnosis, treatment, education, and medication management [1].

Patients with substance use disorder, in particular, are having great benefit from telepsychiatry. Substance users were at a risk of developing coronavirus disease 2019 (COVID-19) infection during the pandemic. Maintaining proper social distance while providing much-needed care was necessary, and telepsychiatry was a solution [2,3].

Although dependence is a chronic disorder characterized by a persistent and intense urge to use illicit drugs, we have included studies containing opioids, alcohol, and tobacco use. The United States has a massive problem with alcohol abuse. About 95,000 lives have been lost due to excessive alcohol use. In 2019, around 414,000 12-17-year-olds were dependent or addicted to alcohol. Only about 7% of persons with alcohol use disorder (AUD) obtained help for it. Alcohol use is a significant problem in rural communities due to limited access to and availability of health and behavioral services. According to current statistics, there is a dire need for treating addiction patients [2,3].

Cigarette smoking is still the leading avoidable cause of mortality worldwide, accounting for more than 480,000 annual deaths. In 2020, roughly 13 out of every 100 individuals in the United States who were 18 or older were still regular cigarette smokers. According to these data, around 30.8% of adult Americans are active smokers. Diseases caused by smoking affect about 16 million people in the United States [4].

Reports indicated increased mortality from drug overdoses during the epidemic. Around 841,000 people in the United States died from drug overdose between 2000 and 2019, according to a survey conducted by the Substance Abuse and Mental Health Services Administration (SAMHSA). SAMHSA reports that in 2022, the funding for the Substance Abuse Prevention and Treatment Block Grant (SABG) was increased to $3.5 billion, which is $1.7 billion higher than 2021, and will be used to enhance the nationwide implementation of evidence-based treatment and prevention programs for people, families, and communities. SAMHSA was able to help an additional 2.1 million people in 2022 because of these funds [5].

Telepsychiatry acts as a bridge between clinicians and patients with substance use disorders (SUD); this can be achieved via calls and video conferencing. Since substance use disorder is a chronic but curable condition, physicians must maintain contact with patients for longer periods. Addiction is characterized by a compulsive drive to use substances despite adverse effects. Through telemedicine, the patient and clinician can maintain a continuous line of communication for extended durations. Patients usually decide whether to refrain from substance use within the context of therapy, where experts are not readily available. Telemedicine might be an immediate resource and consultant in such situations [6].

Increased accessibility to addiction treatment services is achieved through telepsychiatry by lowering numerous barriers such as: (1) Patients in outlying areas no longer have to travel far to receive addiction treatment, as they can do so from the comfort of their own homes or at a nearby medical facility, (2) Patients may get help for their addiction without worrying about the social consequences of going to a specialized treatment facility, (3) Telepsychiatry helps in the reduction of leaves or absences from work, nd (4) Telepsychiatry helps in reducing transportation time and permits flexibility in scheduling appointments. There is a considerable requirement for outpatient follow-up and medical management in numerous addiction health conditions. With telepsychiatry reducing the patient waiting time, cost, the chance of contracting a new illness, and the need for hospitalization, all these could help increase telepsychiatry utilization [1,6].

There is growing evidence that telemedicine can improve access to treatment for SUD. Despite studies showing a significant (approximately 20-fold) growth in the use of telehealth for SUD from 2010 to 2017, telehealth has represented just a tiny percentage of overall telepsychiatry visits in SUD-related treatment [7].

Despite the increase in the use of telepsychiatry during COVID-19, there are specific questions to be considered, such as whether the patient will accept telepsychiatry use, and if accepted, what would the prognosis of the patient be, and whether the treatment via telepsychiatry would continue at the same rate after the pandemic. The prognosis of patients with SUD who use telepsychiatry is analyzed, along with the effects of the COVID-19 epidemic on telepsychiatry, the challenges experienced by both clinicians and patients, and the solutions offered to address these issues.

## Review

Methods

Source of Data Collection

This systematic review followed the Preferred Reporting Items for Systematic Reviews and Meta-Analyses (PRISMA) criteria [8], using the PubMed and Google Scholar databases in a comprehensive data search. All publications published between January 1, 2010, and June 12, 2022, were considered for this study. PubMed and Google Scholar were searched using keywords and the medical subject heading (MeSH) method to get as many relevant articles as feasible. Table 1 shows a list of search terms used to find relevant articles in PubMed.

**Table 1:** Keywords used for PubMed search

Keywords	Database	Number of Studies
telepsychiatry and addiction treatment	PubMed	44 results
telemedicine and substance use disorder treatment	PubMed	507 results
telepsychiatry and substance use disorder treatment	PubMed	18 results
telemedicine and addiction treatment	PubMed	417 results
telepsychiatry and addiction therapy	PubMed	34 results

We used the following MeSH strategy to search for relevant papers: (Addiction (''Addiction Medicine''[Majr])) AND (Telepsychiatry OR Telemedicine (''Telemedicine''[Majr]) AND (''Substance-Related Disorders/education''[Mesh] OR ''Substance-Related Disorders/prevention and control''[Mesh] OR ''Substance-Related Disorders/therapy''[Mesh]).

In addition, we used snowball searching to sift through the citations of included papers for more relevant results.

Inclusion and Exclusion Criteria

Articles published in English between 2010 and 2022 were eligible for inclusion. No limitations were placed on the study types used including randomized controlled trials, case reports, original articles, and standard reviews. Articles were excluded if they did not have an available abstract, were not finished, were never published, or were published before 2010.

Screening and Quality/Bias Assessment

Multiple quality evaluation techniques were employed, including the Assessment of Multiple Systematic Reviews (AMSTAR) tool and PRISMA checklist for systematic reviews, the Cochrane risk of bias tool for randomized controlled trials, the Newcastle-Ottawa scale checklist for observational studies, the Joanna Briggs check tool for case reports, and the Scale for the Assessment of Narrative Review Articles (SANRA) for papers without a clearly defined methods section.

Results

**Figure 1:**
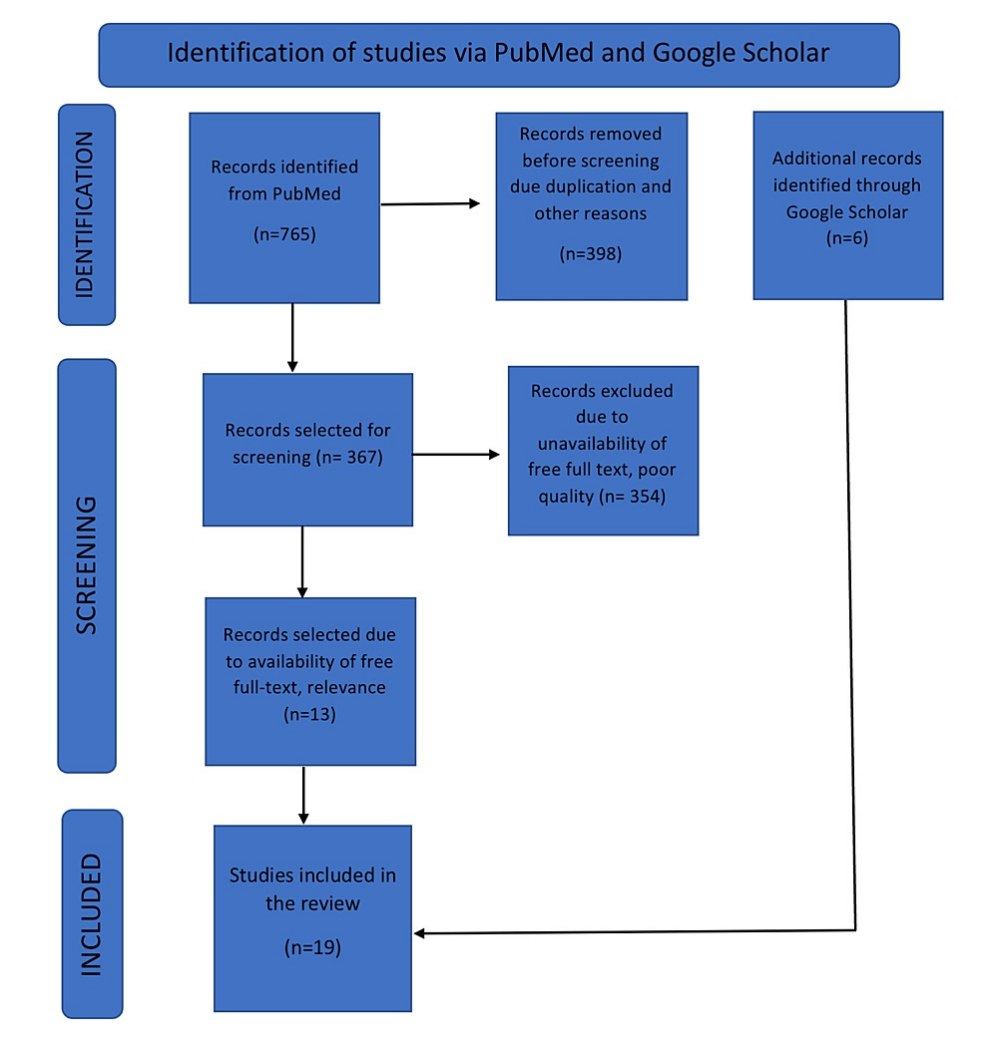
Flowchart with the description of sorting process for paper selection

A total of 765 potentially eligible records were extracted in the initial data retrieval process. During the screening process, 398 records were eliminated due to duplication. The remaining 367 studies were reviewed and 354 articles were excluded for not meeting inclusion criteria. Additionally, six more studies were found on Google Scholar. Finally, 19 studies were selected in the systemic review. The PRISMA process describes the search process and the identification of studies is shown in Figure 1.

Discussion

Although telemedicine was first used in 1879, as described by The Lancet, the application of telemedicine to the field of psychiatry, or telepsychiatry, was first introduced in the 1950s at the Nebraska Psychiatric Institute via videoconference. Through technological communication, telepsychiatry offers consults, treatment, and therapy, through synchronous and asynchronous telepsychiatry sessions. Synchronous telepsychiatry entails a direct communication channel between psychiatrist and patient via live voice or video conferencing. In contrast, asynchronous telepsychiatry is a recorded semi-structured psychiatric patient interview performed by a trained clinician in a primary care clinic that is sent to a psychiatrist for evaluation. The asynchronous approach allows the patient's primary care physician to carry out treatment recommendations [1].

Telepsychiatry is currently being utilized by all the different branches of psychiatry, including the treatment and management of addiction [1].

Addiction is a chronic disease, and patients often need regular care or intensive treatment for a substantial period before they can effectively abstain from the targeted substance [9]. Addiction in the form of alcohol, tobacco, opioids, and other drugs has various treatment methods, such as behavioral counseling or therapy, medication management, use of a medical tool or software to alleviate withdrawal symptoms or teach a new skill, evaluation and treatment of co-occurring mental health disorders, and relapse prevention with extended follow-up.

Thanks to telepsychiatry, these treatment methods can be delivered to the patient either via videoconferencing calls or telephone calls in situations where patients and their psychiatrists cannot meet [10].

Telepsychiatry During the COVID-19 Pandemic

The wide availability of smartphones in recent years has led to a rise in the usage of real-time videoconferencing for medical purposes [7].

Prior to the pandemic between 2016-2019, a survey with a sample including an average of 12,334 treatment facilities reported that telemedicine use grew from 13.5% in 2016 to 17.4% in 2019. Telemedicine use in the treatment of SUD patients has shown a promising use before COVID-19, but the development was slow [11].

Addiction treatment programs expeditiously increased with the start of the pandemic and were delivered through telehealth despite limited information to guide their delivery. Due to the travel and movement limitations enforced by governments throughout the globe due to the COVID-19 epidemic, telemedicine services have been rapidly adopted as the first line of treatment for addiction. The use of telepsychiatry during COVID-19 has provided numerous benefits, including allowing immunocompromised patients to receive much-needed psychiatric care and lowering the risk of contracting the virus for both healthcare providers and patients. Without physically seeing their patients, psychiatrists might still give adequate treatment, and patients who have tested positive for COVID-19 may get therapy through telepsychiatry [12].

Controlled medications like buprenorphine and methadone needed a physical inspection by the prescribing doctor before the COVID-19 epidemic under the Ryan Haight Online Pharmacy Protection Act of 2008 and Drug Enforcement Administration (DEA) rules. New federal and state laws for Medicine for Opioid Use Disorder (MOUD) therapy have been implemented in response to the COVID-19 epidemic. Because of the declaration of a national emergency caused by the COVID-19 outbreak, the DEA and other agencies, including the Substance Abuse and Mental Health Services Administration (SAMHSA) and the Centers for Medicare & Medicaid Services (CMS), have relaxed regulations on Health Insurance Portability and Accountability Act (HIPPA) compliance, Medicare coverage for audio-only visits, and the prescribed controlled substance. Because of these revamped regulations, the clinician could prescribe controlled substances such as buprenorphine via virtual counseling services even without the clinician's first compulsory in-person visit by the patient for examination. Even though these modified regulations may be for use only during the public health emergency, they have drawn higher attention toward telemedicine. Although both clinicians and patients have gained skills and experience with telehealth out of necessity during the pandemic, the use of it after the pandemic depends upon several factors, such as whether the revamped prescription, licensing, and reimbursement regulations will continue or not, and whether a patient will prefer in-person counseling or telepsychiatry for treatment [13].

Prognosis of SUD Patients with Telepsychiatry

Methods to measure the prognosis of SUD patients with telepsychiatry depend upon multiple factors, such as (a) Whether the symptoms will get better, become worse (and how quickly), or stay the same over time, (b) Health-related factors that affect one's quality of life, such as the risk of developing complications and the severity of existing health problems, (c) Period of time from abstinence, (d) Relapse prevention, and (e) Available therapies [14].

The normal progression of the illness, the individual's physical and psychological state, the available therapies, and other considerations are all considered when making a prognosis. A full prognosis will contain details like how long it's projected to last, how much better the function is likely to become, and how it'll change over time, whether gradually, in spurts, or all at once [14].

Seven publications were included to study the prognosis of addiction patients using telepsychiatry/telemedicine for treatment. Four of the seven included publications dealt with opioid use disorder, another dealt with alcoholism, one dealt with tobacco, and the final one was on SUD. Table 2 represents the studies used to review the prognosis of addiction patients with the use of telepsychiatry.

**Table 2:** Studies used to review the prognosis of addiction patients with telepsychiatry use IOP: intensive outpatient therapy; MAT: medication-assisted treatment

Publication	Year	Type of study	Population	Intervention	Key Findings
Gliske et al., 2022 [15]	2022	Observational study	Treatment for drug use disorders is provided to patients with the disorder (N=3642 patients).	Those currently being treated for drug use disorders might benefit from intensive outpatient care in one of three delivery modes: traditional, hybrid, or digital.	Patients treated primarily using a hybrid approach (telehealth plus in-person therapy) were more likely to complete their treatment program than those treated primarily either in-person or virtual intensive outpatient therapy (IOP).
Riedel et al., 2021 [16]	2021	Observational study	Clinicians treating patients with opioid use disorder (N=602).	Treating opioid use disorder Patients with telemedicine.	Telemedicine care they have provided has been as effective as in-person care.
Morin et al., 2021 [17]	2021	Retrospective Cohort study	Patient with opioid use disorder receiving opioid agonist treatment (N=55,924).	Patients receiving opioid agonist treatment via telemedicine, in-person, and a mix of telemedicine and in-person.	When it comes to opioid-related deaths, Emergency related visits, and patient retention, telemedicine is on par with in-person treatment.
Eibl et al., 2017 [18]	2017	Retrospective observational study.	Patients with opioid use disorder receiving medications for opioid use disorder (N=3,733).	Medication treatment for opiate use disorder by a medical professional.	The retention rates of patients treated primarily via telehealth or a hybrid of telehealth and in-person therapy were higher than those of patients treated primarily via in-person therapy.
Zheng et al., 2017 [19]	2017	Retrospective Cohort study	Buprenorphine MAT programs for the treatment of opioid use disorder patients (N=100).	Physician-delivered opioid use disorder buprenorphine Medication-assisted treatment (MAT) remotely via telepsychiatry or in person.	We found no statistically significant difference between face-to-face MAT therapy and telepsychiatry buprenorphine MAT intervention in our Comprehensive Opioid Addiction Treatment (COAT) model for those with opioid use disorder.
Tarp et al., 2017 [20]	2017	Randomized control trial	Patients with alcohol use disorder (N=71).	Alcohol use disorder treatment in person or in person combined with videoconferencing.	Patients may be more likely to complete their treatment sessions if they have the option of attending through videoconference rather than dropping out completely.
Carlson et al., 2012 [21]	2012	Retrospective Cohort study	Patients with smoking disorders receiving smoking cessation interventions (N=554).	Smoking cessation interventions were provided to the patients via telehealth videoconferencing and in-person visits.	Patients treated for smoking cessation produced similar Quit rates when treated with telehealth videoconferencing technology as well as in-person treatment.

A longitudinal research conducted between January 2020 and March 2021 on 3642 patients with SUD found no significant differences in continuous abstinence, overall quality of life, financial well-being, psychological well-being, and confidence in one's ability to stay sober when treated by various health formats like in-person care only (n=957, 26.3%), hybrid, in-person and virtual care (n=541, 14.9%), and virtuosic care (n=2144, 58.9%) [15]. This study shows telepsychiatry is equally effective in the treatment and prognosis of SUD patients when compared to in-person treatment.

One of the studies on opioid prognosis included 3733 patients with opioid use disorder who were treated with opioid agonist therapy through in-person, telemedicine, and mixed (in-person and telemedicine) approaches [18]. The research found that telemedicine patients had a higher retention rate than in-person patients (n = 1590) when it came to continuing their treatment. Telemedicine patients had a retention rate of 50% after one year, while in-person patients maintained a rate of 39%. At 47%, the mixed group's retention rate was higher than that of the in-person group (n = 418). In contrast, three other studies, which included 602 patients, 55,924 patients, and 100 patients with opioid use disorder, where all of them received treatment via in-person, telemedicine, and mixed methods, revealed that telemedicine was equally effective in comparison with in-person treatment over retention rates or abstinence [16,17,19].

Treatment in-person (face-to-face) and treatment in-person with add-on intervention (optional video conferencing) were compared in a study of 71 patients with AUD, and the results suggest that providing patients with the option to participate in videoconferencing may reduce early dropouts and increase the length of treatment [20].

Another study was conducted on 554 patients with a smoking disorder, where smoking cessation interventions were provided in rural areas via telehealth videoconferencing technology and in-person treatment to the patients living in urban locations. The study revealed that quit rates were equal when treated with either treatment method [21]. 

The overall studies, when combined, gave an overview that telepsychiatry/telemedicine treatment in the prognosis of addiction patients is similar to in-person treatment [15,17,19-21]. In contrast, two studies have mentioned that telepsychiatry, combined with in-person treatment, produced higher retention rates and decreased premature dropouts [16,18].

Difficulties in Practicing Telepsychiatry

Telepsychiatry's use in treating patients with SUD can have many benefits for the patient and clinician, but it also presents particular challenges.

Difficulties Faced by Clinicians: Cultural and communication gaps and building rapport with the patient are some problems clinicians face in various parts of the world, impacting clinician interaction and treatment with patients in the field of telepsychiatry. Language is another barrier in several countries where clinicians face difficulty communicating with patients when they do not speak or know the same language. Such a problem arises in countries where people with different ethnic or races live together or in countries with high immigration. One such example is the United States, where people from other parts of the world immigrate, simultaneously resulting in cultural gaps and language problems [22].

Clinicians also face difficulties during their first encounter with telepsychiatry due to their lack of technological training or experience with electronic devices. This can be challenging during patient documentation and identification and during the use of videoconferencing software [16]. A clinician could face difficulties while treating patients due to a lack of proper internet connectivity or technical glitches in the software/hardware, poor audio/visual quality, audio/visual lag, and at times the lack of electronic devices/instruments required for the use of telepsychiatry for treatment of addiction patients [16].

Obtaining vital signs and the necessary physical examination also present a challenge during telepsychiatry [1]. Except for individuals in recognized healthcare settings, federal law forbids restricted medication prescription. Buprenorphine may now be started on the phone according to new laws. However, methadone, another life-saving drug for opioid use disorder, is still subject to strict restrictions and requires an initial in-person visit [23].

Most jurisdictions need doctors to have active licenses in the states where their patients live. Only 14 states have given out-of-state doctors conditional or telemedicine licenses. It is clear that the time and money required to maintain numerous licenses, together with the complexity of legislation that changes across states, places a considerable strain on doctors since 93.5% of telemental health appointments in 2014 occurred in the same state of both the patient and practitioner. A recent bill that would have required federal health plan providers only to be licensed in the state where they practice but would have allowed them to treat patients from any state in the country died in committee [23]. In several states, obstacles and problems with payment regulations persist [23].

Patient denial in the form of distraction or forgetfulness to avoid discussing their treatment is one of the difficulties faced by clinicians while using telepsychiatry to treat addiction patients. Disturbance and inappropriate behavior by the patient at the time of treatment are other difficulties/problems faced by the clinician [24]. 

Difficulties Faced by Patients: Language and culture gaps act as a barrier even for patients, when living in countries with a mixed population. This causes difficulty for the patient to communicate or express his/her complaints with the physician [22].

Affordability in accessing the technological equipment could be challenging for certain patients from poor socioeconomic backgrounds. Patients living in rural areas face issues with network coverage. Lack of proper network coverage/connection disrupts the ongoing telepsychiatric videoconferencing session between the patient and psychiatrist, thereby affecting the output [16].

Telepsychiatry may be challenging for the elderly and the technologically illiterate if they lack the skills necessary to participate in video sessions. These patients could require an audio call, and they might have trouble opening emailed documents or filling emailed prescriptions. Besides, most elderly patients live alone and may not have access to drugs, making it difficult to get medication, even after obtaining therapy and prescriptions online [1].

Measures to Improve These Difficulties

Collaboration with local professionals in patient care may assist in establishing rapport with the patient, offer insight into community resources, and bridge any communication gaps that may exist [23].

Healthcare providers and patients may benefit from digital literacy training in telepsychiatry by improving patient identification and documentation skills. The use of telepsychiatry for the treatment of addiction may increase if the necessary telepsychiatry technology were made available to doctors and patients [25]. 

The COVID-19 pandemic has resulted in new federal and state regulations governing medication for opioid use disorder MOUD) treatment such as the clinician could prescribe controlled substances like buprenorphine via virtual counseling services even without the first compulsory in-person visit by the patient for examination by the clinician. CMS has eased regulations on HIPPA compliance and Medicare coverage for audio-only visits. The continuation of such revamped regulations could reduce the difficulties clinicians and patients face while using telepsychiatry [13].

The solutions proposed for licensure issues include establishing national licensure, making the referring physician responsible for the patient and using the consulting telepsychiatrist's opinion as a recommendation, or deciding that the patient is being ''electronically transmitted'' to the consultant's state and thus the consultant is not required to be licensed in the patient's state [23].

Although online therapy has many benefits, therapists must be mindful of contextual factors like location and physical proximity when working with clients. As the therapeutic atmosphere must be taken seriously, the setting must be set up like a clinic rather than a person's home. Furthermore, the treatment has to be conducted in a timely and expert manner. Even if the therapist works from home, they still cannot have any noise or interruptions from loved ones during sessions. This could promote patient denial and even disturbance during treatment [23].

To enable telepsychiatry use by healthcare providers and patients at a higher level, the healthcare systems and state policymakers should first invest in telehealth infrastructures such as HIPAA-compliant technology and equipment. Such a move could increase the availability of devices required for telepsychiatry use. Devices with better video and audio quality help improve the telepsychiatry delivered addiction treatment [26].

The broadband infrastructure required for video-based telepsychiatry visits is in more than 40% shortage in major rural counties of the United States. The expansion of broadband cable by the telecommunications department has increased internet access. The beginning of the 5G cellular network rollout across several parts of the world would improve the range and quality of wireless networks and increase network speed, helping the rural population [26].

Limitations

Due to the small study numbers, this research could not draw any firm conclusions on the effect of telepsychiatry on the long-term outcomes of those struggling with addiction. We only looked at data from 2010 onwards, so some essential studies may have been left unnoticed. Because of the confined search technique, several studies relevant to the review topic may have been missed. Animal studies and research published in other languages were excluded from this study.

## Conclusions

The COVID-19 pandemic, along with increased use of technology and revamped regulations, has forced telepsychiatry utilization in the treatment of addiction patients. Although telepsychiatry use provides certain advantages, there are some disadvantages/difficulties present at the same time, which can be overcome by certain methods. Continuation of the updated prescription, licensing, and reimbursement laws even after the pandemic would benefit telepsychiatry utilization. The overall results of this review indicate that the prognosis of addiction patients with telepsychiatry is similar to the in-person treatment of addiction patients. Nonetheless, some studies have mentioned that the combination of telepsychiatry with in-person treatment would deliver higher treatment results than individual methods. Even though telepsychiatry use during the pandemic has unprecedentedly increased, its use after the pandemic will still depend upon several factors such as government regulations, secure and encrypted platforms to create more doctor-patient confidence, and physician licensing.
